# Supplementary material for: Revealing the etching process of water-soluble Au25 nanoclusters at the molecular level
Source: Nat Commun. 2021 May 28;12:3212. doi: 10.1038/s41467-021-23568-0 (PMC8163824; doi:10.1038/s41467-021-23568-0)
Supplement: Supplementary file 1 — Supplementary information. [file 41467_2021_23568_MOESM1_ESM.pdf]

Supplementary Information

Revealing the etching process of water-soluble Au_{25} nanoclusters at the molecular level

Yitao Cao,[1] Tongyu Liu,[2] Tiankai Chen,[1] Bihan Zhang,[1,3] De-en Jiang,[2] Jianping Xie[1,3,*]

1. Department of Chemical and Biomolecular Engineering, National University of Singapore, 4 Engineering Drive 4, Singapore 117585, Singapore
2. Department of Chemistry, University of California, Riverside, California 92521, United States
3. Joint School of National University of Singapore and Tianjin University, International Campus of Tianjin University, Binhai New City, Fuzhou, China 350207

<u>This file includes:</u>

Supplementary Methods

Supplementary Fig. 1-35

Supplementary Table 1-3

Supplementary References

Supplementary Methods

Computational details. The density functional theory (DFT) calculations were performed with the quantum chemistry program Turbomole V6.5.[1] Structural optimizations and calculations of energies were done by DFT within the D3 dispersion correction.[2] The TPSS (Tao, Perdew, Staroverov, and Scuseria) functional was used for electron exchange and correlation,[3] and the def2-TZVPP for orbital and auxiliary basis sets.[4] Gold (Au) uses the viable core potentials with 19 valence electrons including scalar relativistic corrections.[5] The initial structures of $Au_{18}(SCH_3)_{14}$ and $Au_{20}(SCH_3)_{16}$ are constructed by replacing hydrocarbyl in $Au_{18}(SC_6H_{11})_{14}$ and Au_{20}(SPh-*t*-Bu)$_{16}$ with methyl.[6, 7] The structure of $Au_{22}(SCH_3)_{18}$ is developed based on the previous study.[8]

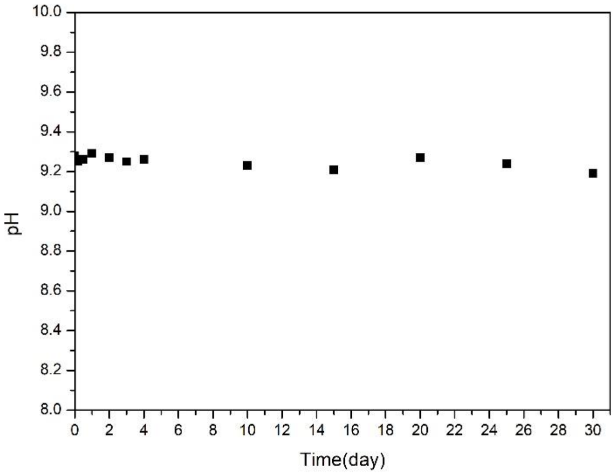

Supplementary Figure 1. pH monitoring in reaction process. pH variation of the reaction solution in the etching process of $Au_{25}(MHA)_{18}$ NCs.

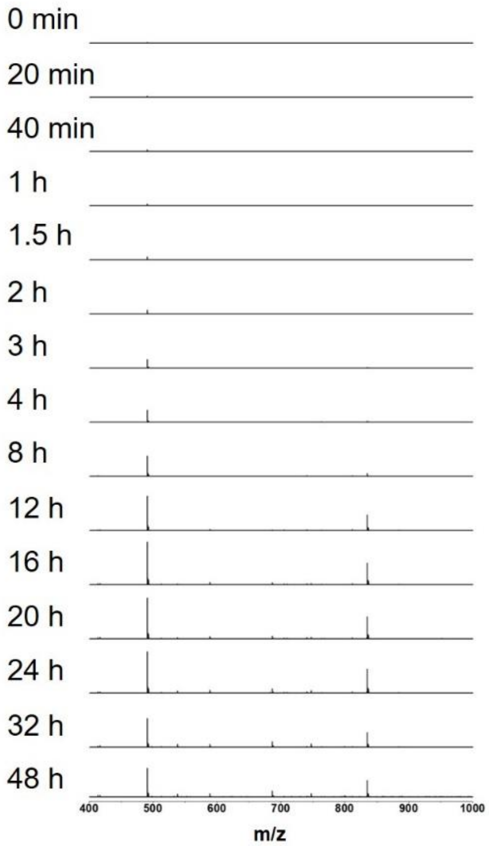

Supplementary Figure 2. Enlarged view of ESI mass spectra in the range of 400-1000 m/z from 0 h to 48 h.

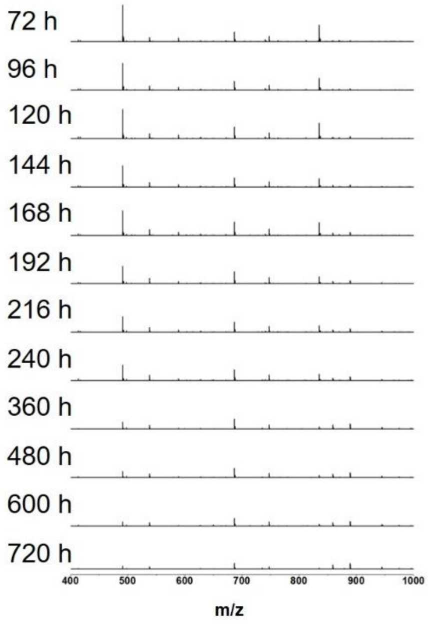

Supplementary Figure 3. Enlarged view of ESI mass spectra in the range of 400-1000 m/z from 72 h to 720 h.

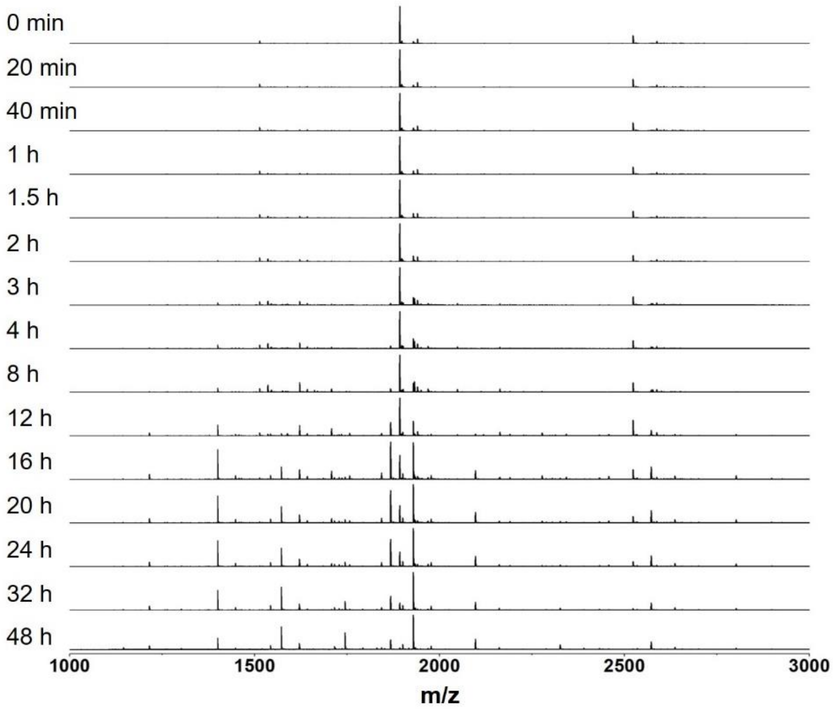

Supplementary Figure 4. Enlarged view of ESI mass spectra in the range of 1000-3000 m/z from 0 h to 48 h.

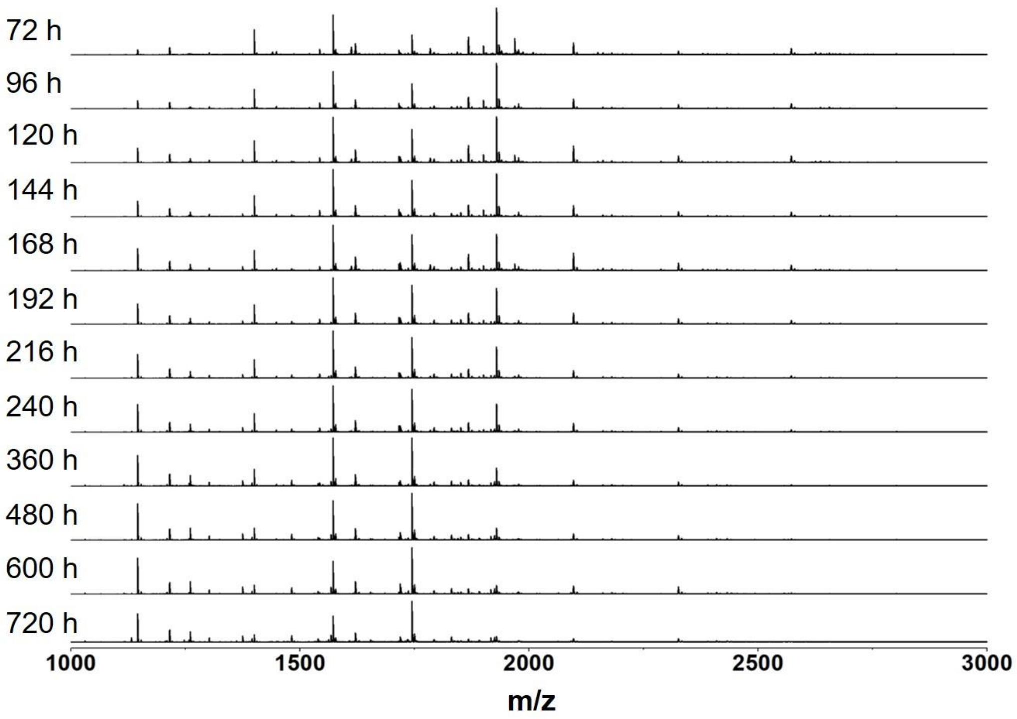

Supplementary Figure 5. Enlarged view of ESI mass spectra in the range of 1000-3000 m/z from 72 h to 720 h.

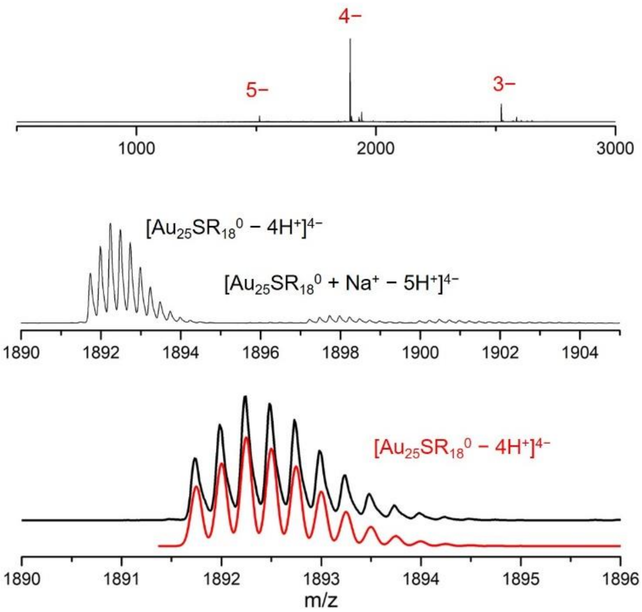

Supplementary Figure 6. ESI mass spectra of purified $Au_{25}SR_{18}$. Both experimental (black line) and simulated (red line) results were shown. The result indicates the main $Au_{25}SR_{18}$ species is neutral in charge.

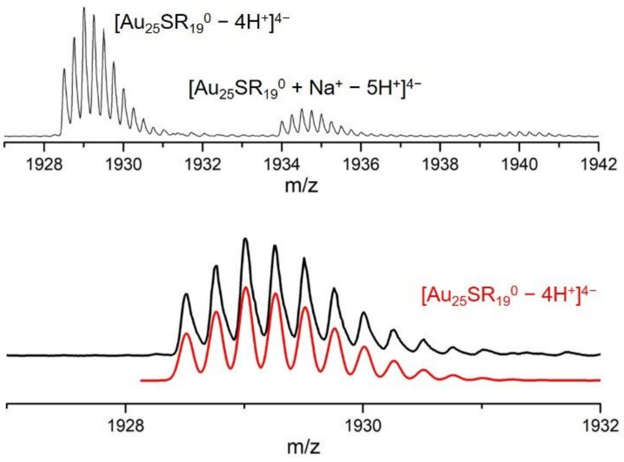

Supplementary Figure 7. ESI mass spectra of $[Au_{25}SR_{19}]^0$. Both experimental (black line) and simulated (red line) results were shown.

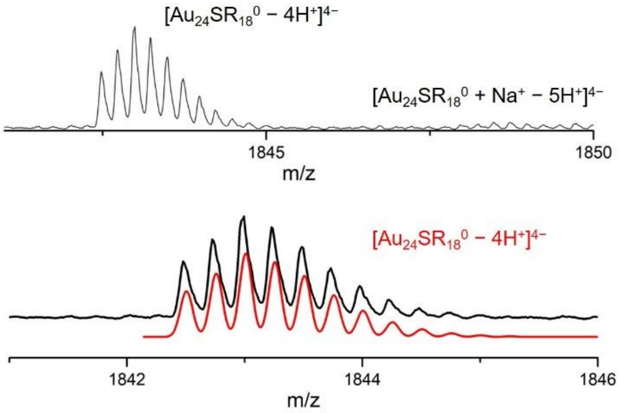

Supplementary Figure 8. ESI mass spectra of $[Au_{24}SR_{18}]^0$. Both experimental (black line) and simulated (red line) results were shown.

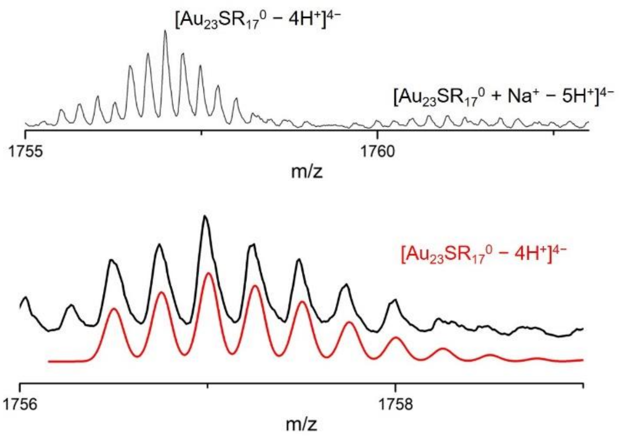

Supplementary Figure 9. ESI mass spectra of $[Au_{23}SR_{17}]^0$. Both experimental (black line) and simulated (red line) results were shown.

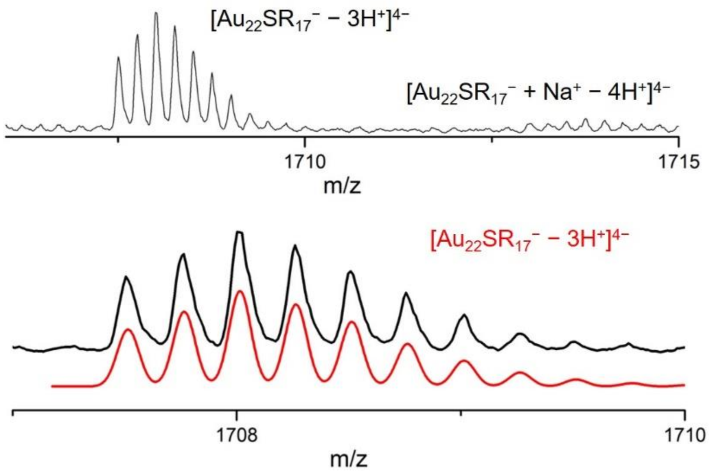

Supplementary Figure 10. ESI mass spectra of $[Au_{22}SR_{17}]^-$. Both experimental (black line) and simulated (red line) results were shown.

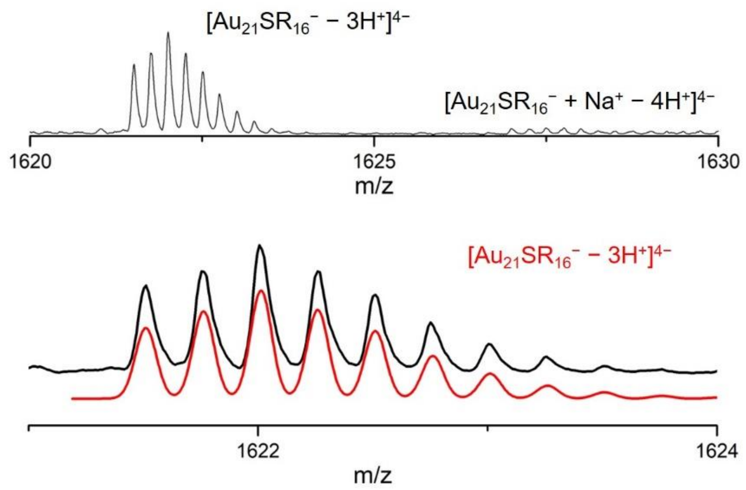

Supplementary Figure 11. ESI mass spectra of $[Au_{21}SR_{16}]^-$. Both experimental (black line) and simulated (red line) results were shown.

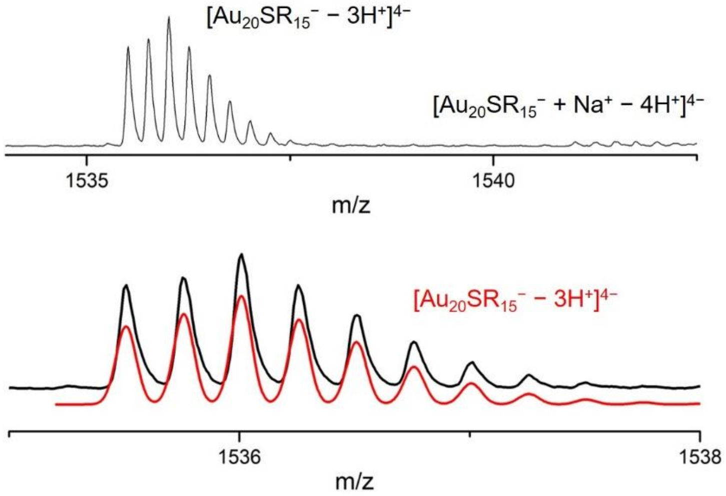

Supplementary Figure 12. ESI mass spectra of $[Au_{20}SR_{15}]^-$. Both experimental (black line) and simulated (red line) results were shown.

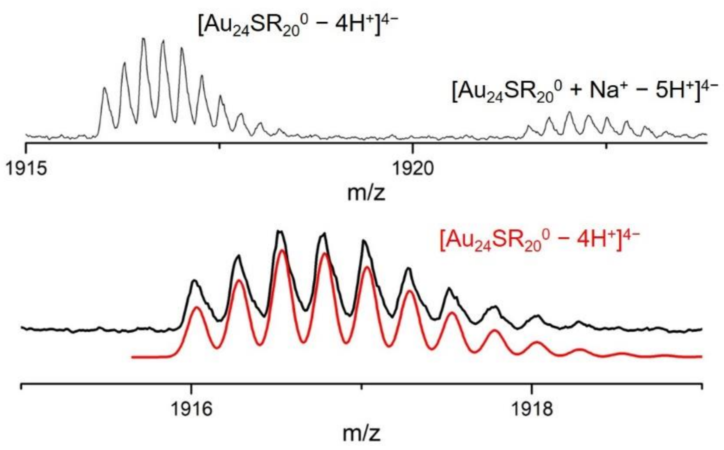

Supplementary Figure 13. ESI mass spectra of $[Au_{24}SR_{20}]^0$. Both experimental (black line) and simulated (red line) results were shown.

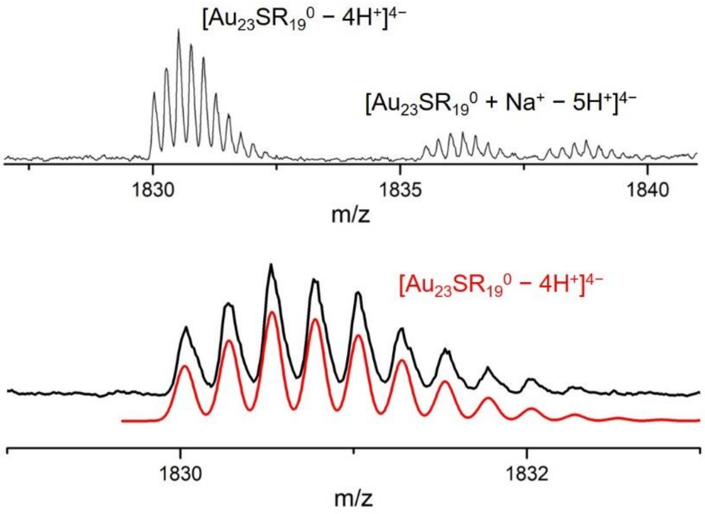

Supplementary Figure 14. ESI mass spectra of $[Au_{23}SR_{19}]^0$. Both experimental (black line) and simulated (red line) results were shown.

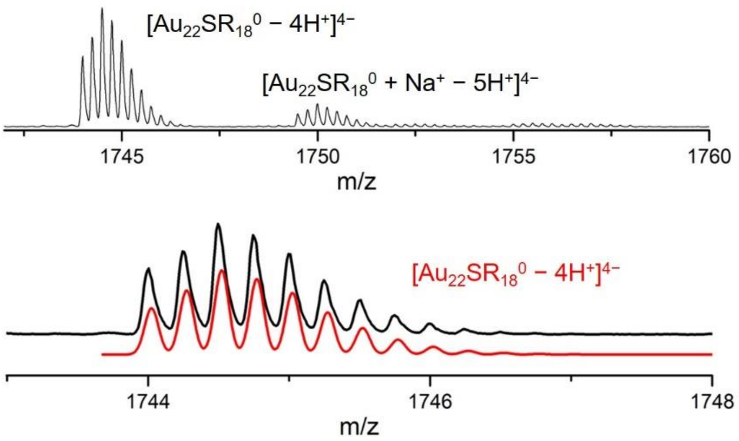

Supplementary Figure 15. ESI mass spectra of $[Au_{22}SR_{18}]^0$. Both experimental (black line) and simulated (red line) results were shown.

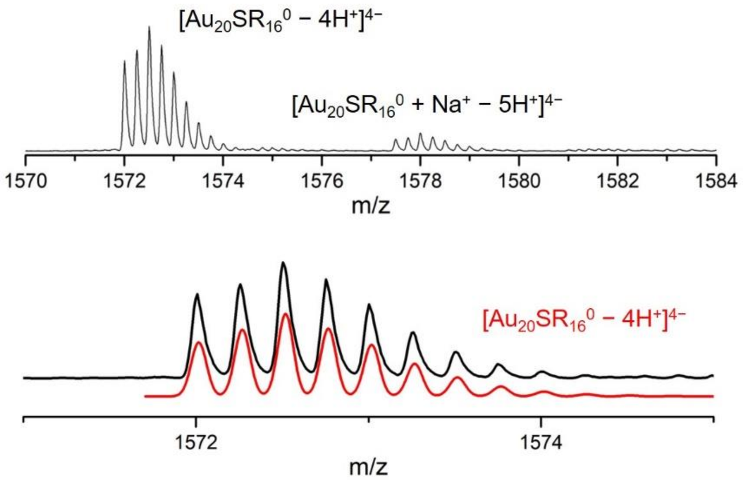

Supplementary Figure 16. ESI mass spectra of $[Au_{20}SR_{16}]^0$. Both experimental (black line) and simulated (red line) results were shown.

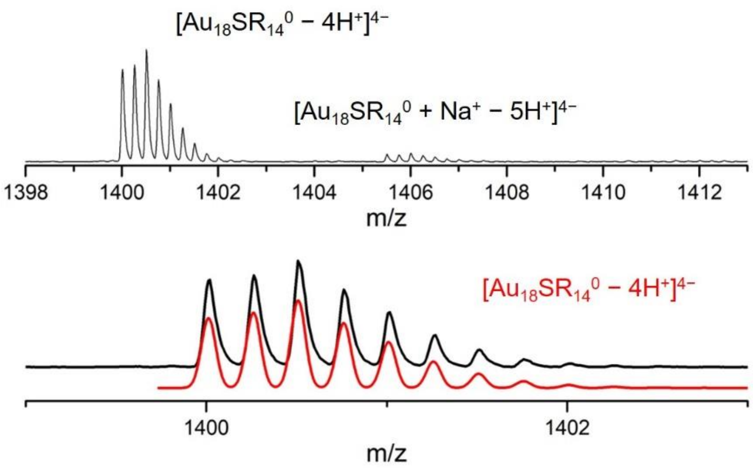

Supplementary Figure 17. ESI mass spectra of $[Au_{18}SR_{14}]^0$. Both experimental (black line) and simulated (red line) results were shown.

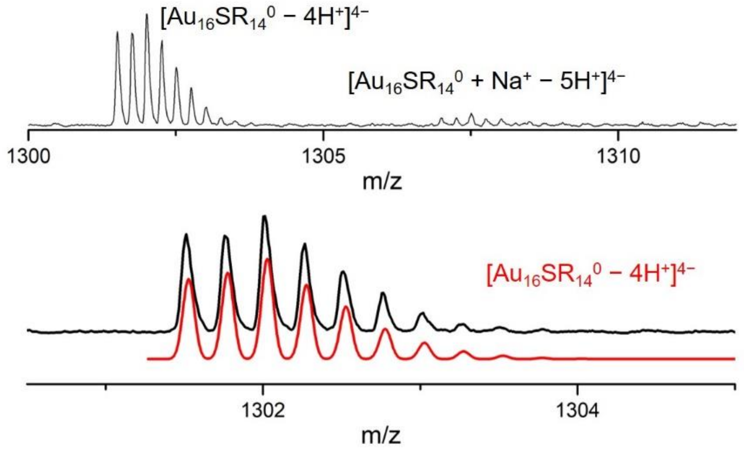

Supplementary Figure 18. ESI mass spectra of $[Au_{16}SR_{14}]^0$. Both experimental (black line) and simulated (red line) results were shown.

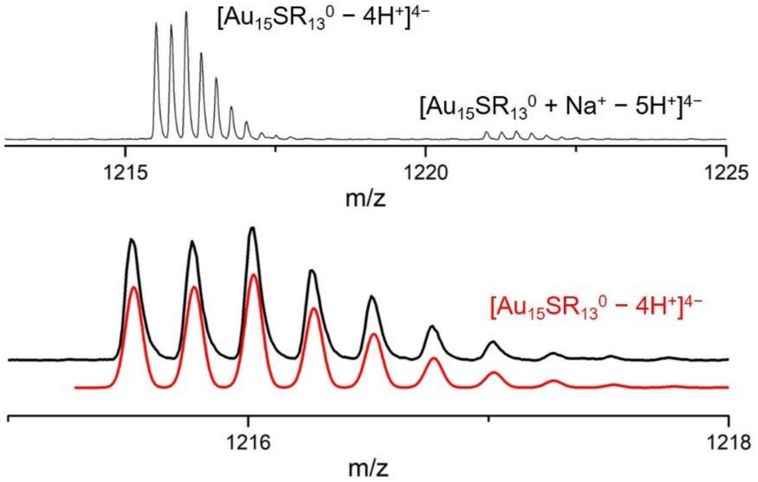

Supplementary Figure 19. ESI mass spectra of $[Au_{15}SR_{13}]^0$. Both experimental (black line) and simulated (red line) results were shown.

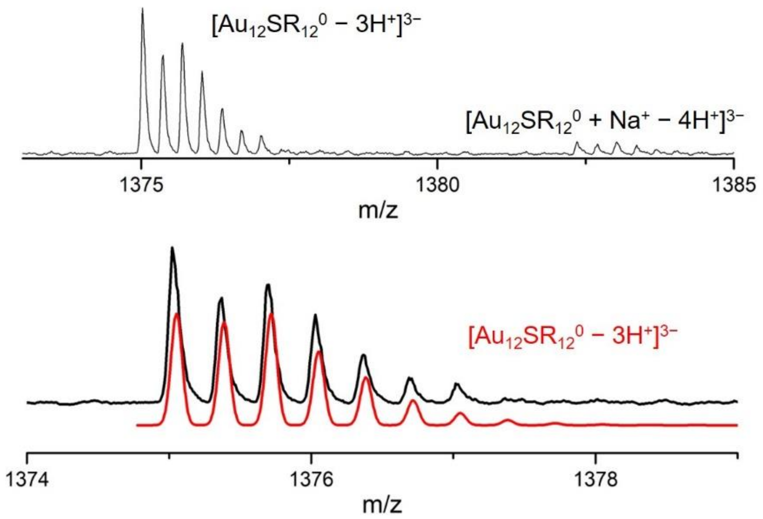

Supplementary Figure 20. ESI mass spectra of $[Au_{12}SR_{12}]^0$ (long-chain complexes). Both experimental (black line) and simulated (red line) results were shown.

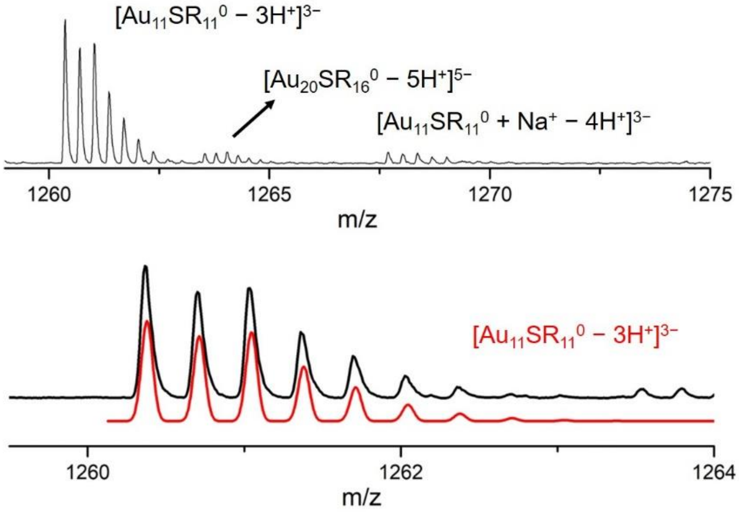

Supplementary Figure 21. ESI mass spectra of $[Au_{11}SR_{11}]^0$ (long-chain complexes). Both experimental (black line) and simulated (red line) results were shown.

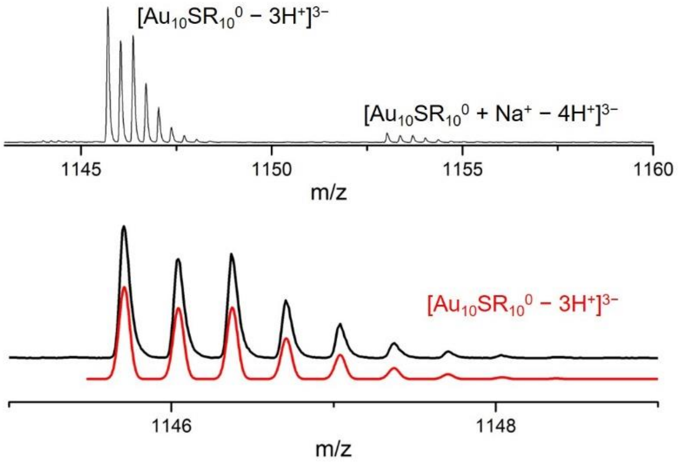

Supplementary Figure 22. ESI mass spectra of $[Au_{10}SR_{10}]^0$ (long-chain complexes). Both experimental (black line) and simulated (red line) results were shown.

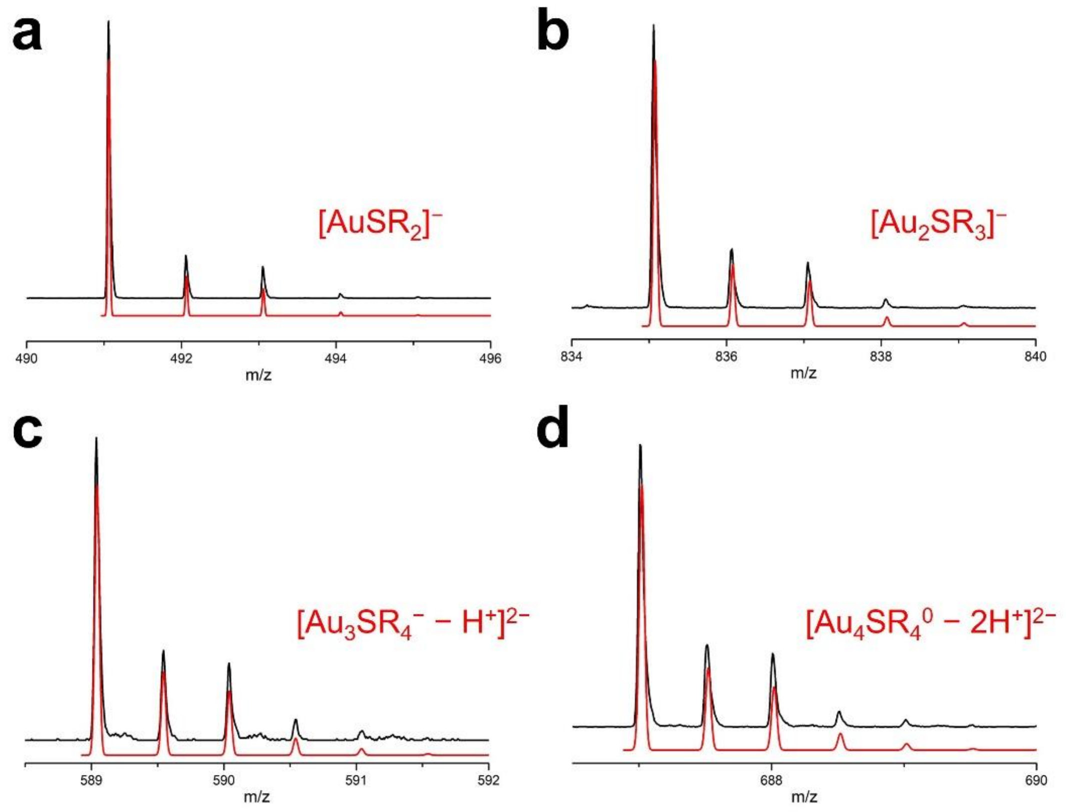

Supplementary Figure 23. ESI mass spectra of small complexes. (a) $[AuSR_2]^-$, (b) $[Au_2SR_3]^-$, (c) $[Au_3SR_4]^-$, and (d) $[Au_4SR_4]^0$. Both experimental (black line) and simulated (red line) results were shown.

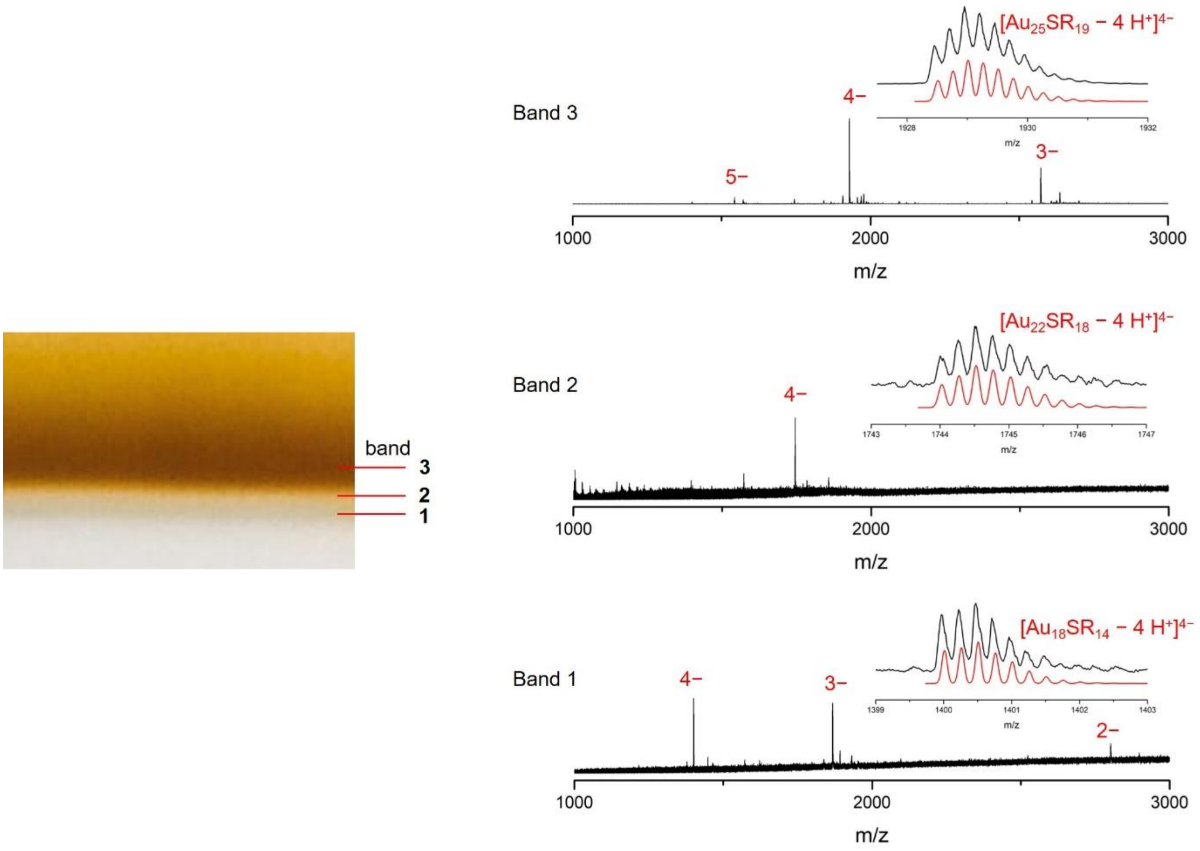

Supplementary Figure 24. PAGE separation of the etching product and the ESI mass spectra of the species in the corresponding bands at the reaction time of 48 h. Three bands were cut from the gel and analyzed by ESI-MS. The ESI mass spectra show signals of $Au_{18}SR_{14}$, $Au_{22}SR_{18}$ and $Au_{25}SR_{19}$ for each band, stepwise from small to large. This result indicates that these species exist in the etching process of $Au_{25}SR_{18}$ NCs, and no in-situ electrochemical reactions occurred in the ESI-MS test. It is worth noting that the resolution of PAGE is not sufficient to separate all species of similar size (band merging is severe), and some species may not be abundant enough to show clear bands. In addition, we cannot ensure the stability of all species during the long electrophoresis process (usually 24 h). Therefore, we did not use PAGE for fulltime screening of all intermediate species.

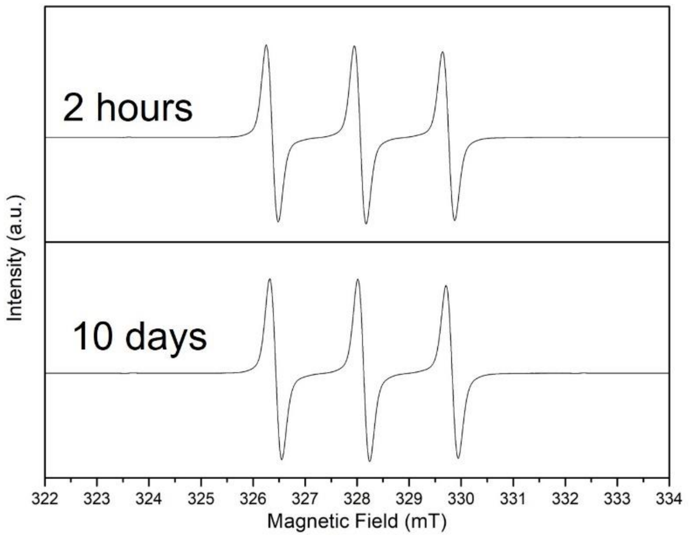

Supplementary Figure 25. EPR capturing of thiol radicals in the reaction process. EPR spectra captured by using 4-OH-TEMPO as trapping reagent in water phase. Two characteristic time points were chosen to show the presence of thiol radicals at different stages of the etching reaction.

$$[Au_{25}SR_{18}]^0 + RS\cdot + 6RS^- \rightarrow [Au_{20}SR_{15}]^- + 5[AuSR_2]^-$$
$$[Au_{25}SR_{18}]^0 + RS\cdot + 5RS^- \rightarrow [Au_{21}SR_{16}]^- + 4[AuSR_2]^-$$
$$[Au_{21}SR_{16}]^- + 2RS\cdot + 2RS^- \rightarrow [Au_{18}SR_{14}]^0 + 3[AuSR_2]^-$$
$$[Au_{21}SR_{16}]^- + 4RS\cdot + 5RS^- \rightarrow [Au_{15}SR_{13}]^0 + 6[AuSR_2]^-$$

Supplementary Figure 26. **Decomposition process mediated by thiol radicals in Stage I.** Decomposition reactions responsible for the formation and consumption of 6 e species were proposed.

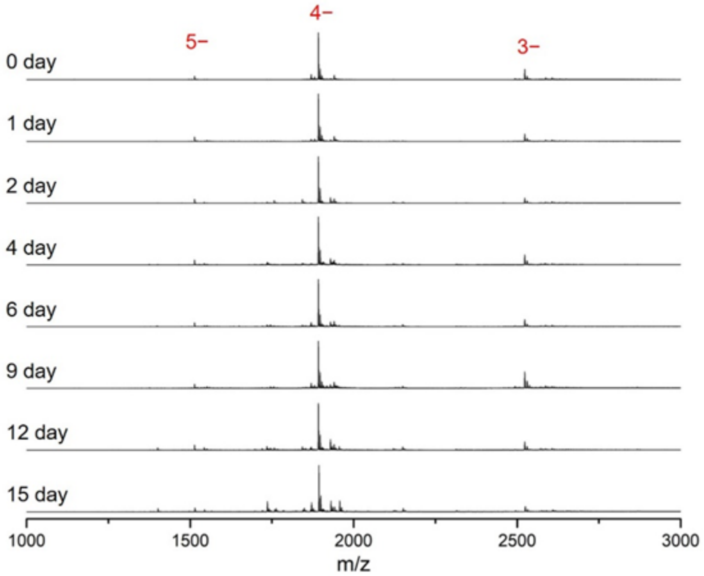

Supplementary Figure 27. Control experiments without excess thiol ligands. ESI mass spectra of $Au_{25}SR_{18}$ NCs without adding excess thiol ligands.

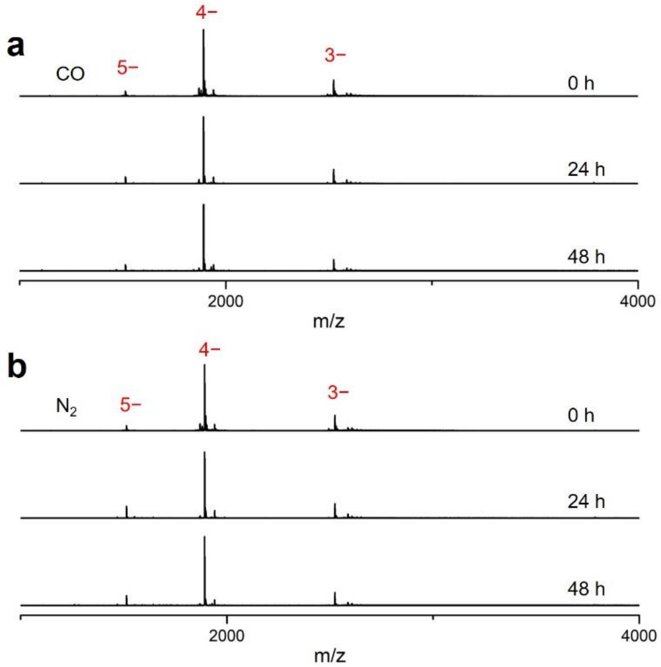

Supplementary Figure 28. Control experiments with reducing/inert gas. ESI mass spectra of the reaction solution saturated by (a) CO and (b) N_2 in 48 hours. The decomposition process has been quenched.

$$[Au_{21}SR_{16}]^{-} + [AuSR_2]^{-} \rightarrow [Au_{22}SR_{17}]^{-} + RS^{-}$$
$$[Au_{22}SR_{17}]^{-} + [AuSR_2]^{-} \rightarrow [Au_{23}SR_{17}]^{0} + 2RS^{-}$$
$$[Au_{23}SR_{17}]^{0} + [AuSR_2]^{-} \rightarrow [Au_{24}SR_{18}]^{0} + RS^{-}$$
$$[Au_{24}SR_{18}]^{0} + [AuSR_2]^{-} \rightarrow [Au_{25}SR_{19}]^{0} + RS^{-}$$

Supplementary Figure 29. Recombination process mediated by isoelectric addition reactions. Only possible reaction routes were shown. The detailed reactions routes need to be confirmed by further experimental efforts and DFT calculations, which are beyond the scope of this work.

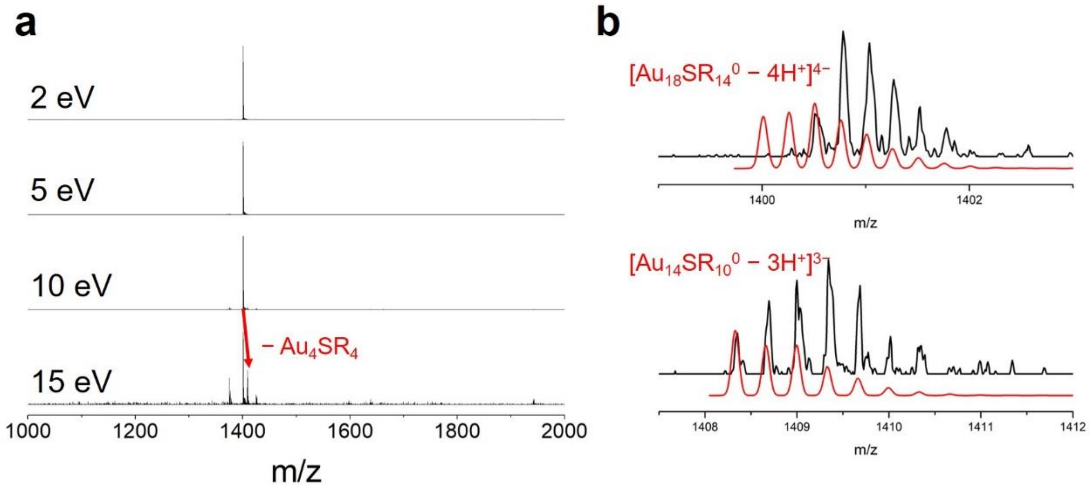

Supplementary Figure 30. MS/MS spectra of $[Au_{18}SR_{14}]^0$. (a) MS/MS spectra of $[Au_{18}SR_{14}]^0$ at different fragmentation energy. (b) Analysis of the corresponding species in MS/MS spectra. The shape change of isotopic patterns is due to the ion filtering in the MS/MS process, while keeping the overall position accurate. Note that the 2nd generation fragmentations and small fragments (e.g., $[Au_4SR_4]^-$) also present in MS/MS spectra and they are not analyzed in detail here.

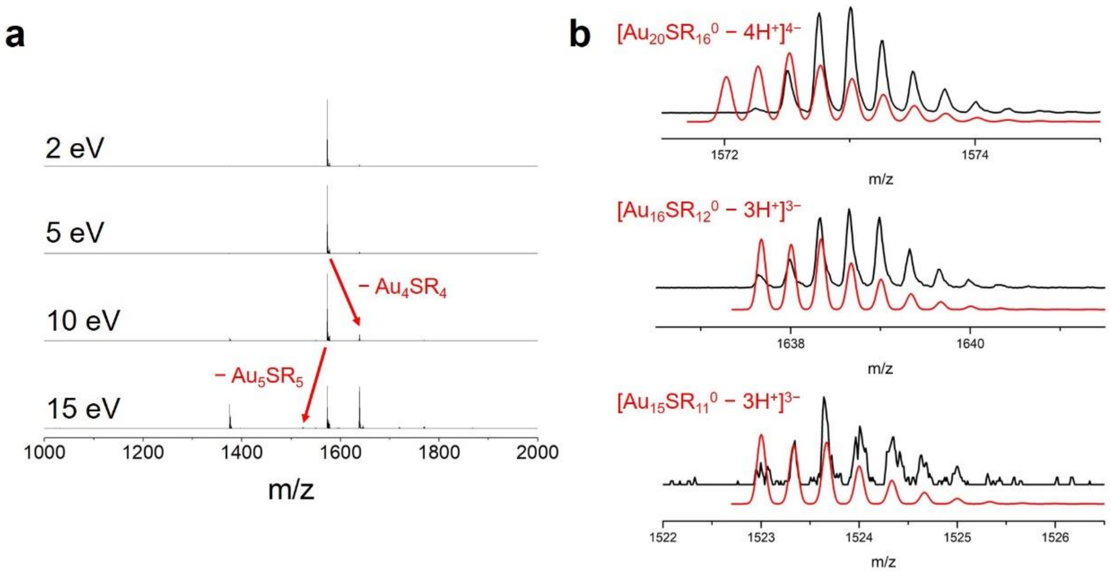

Supplementary Figure 31. MS/MS spectra of $[Au_{20}SR_{16}]^0$. (a) MS/MS spectra of $[Au_{20}SR_{16}]^0$ at different fragmentation energy. (b) Analysis of the corresponding species in MS/MS spectra. The shape change of isotopic patterns is due to the ion filtering in the MS/MS process, while keeping the overall position accurate. Note that the 2nd generation fragmentations and small fragments (e.g., $[Au_4SR_4]^-$) also present in MS/MS spectra and they are not analyzed in detail here.

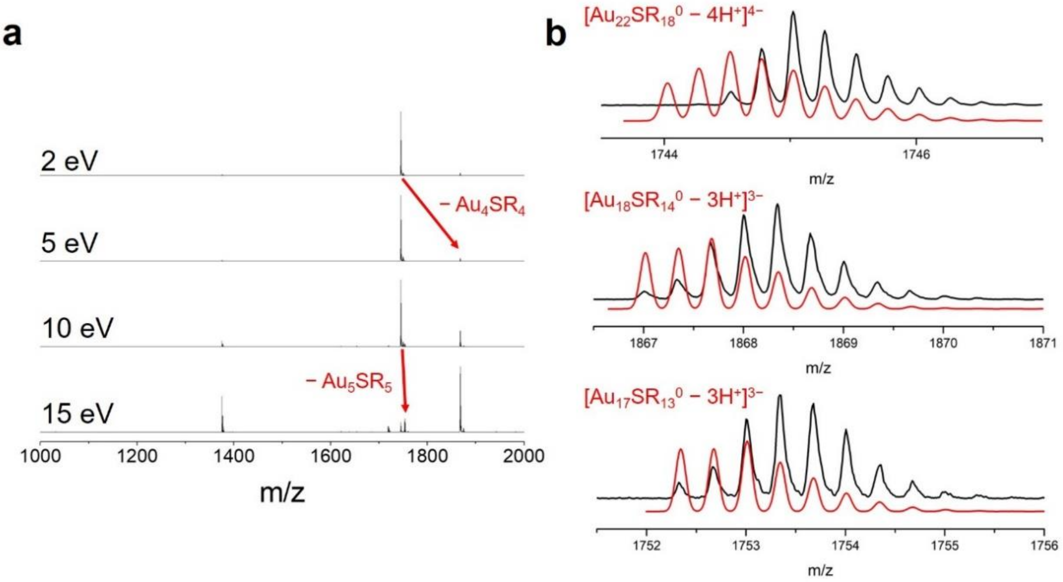

Supplementary Figure 32. MS/MS spectra of $[Au_{22}SR_{18}]^0$. (a) MS/MS spectra of $[Au_{22}SR_{18}]^0$ at different fragmentation energy. (b) Analysis of the corresponding species in MS/MS spectra. The shape change of isotopic patterns is due to the ion filtering in the MS/MS process, while keeping the overall position accurate. Note that the 2nd generation fragmentations and small fragments (e.g., $[Au_4SR_4]^-$) also present in MS/MS spectra and they are not analyzed in detail here.

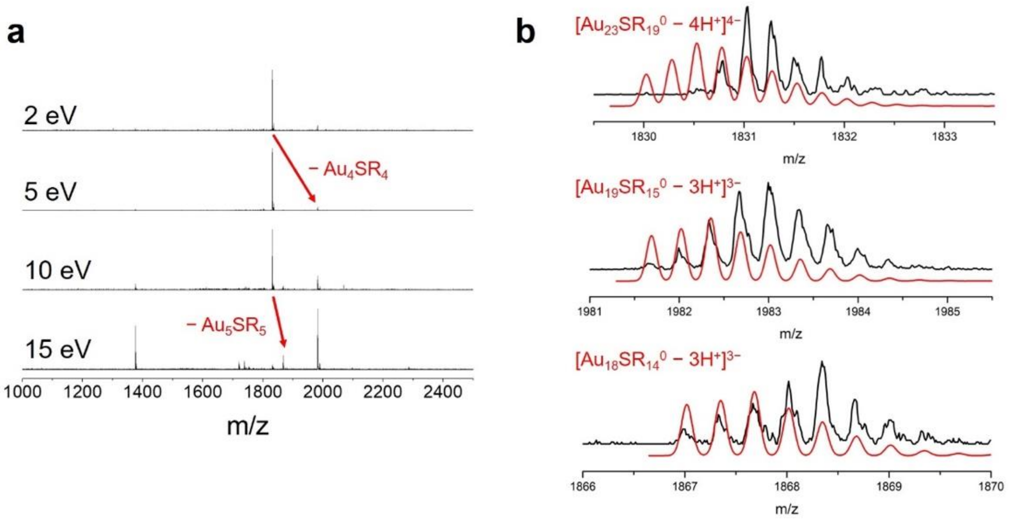

Supplementary Figure 33. MS/MS spectra of $[Au_{23}SR_{19}]^0$. (a) MS/MS spectra of $[Au_{23}SR_{19}]^0$ at different fragmentation energy. (b) Analysis of the corresponding species in MS/MS spectra. The shape change of isotopic patterns is due to the ion filtering in the MS/MS process, while keeping the overall position accurate. Note that the 2nd generation fragmentations and small fragments (e.g., $[Au_4SR_4]^-$) also present in MS/MS spectra and they are not analyzed in detail here.

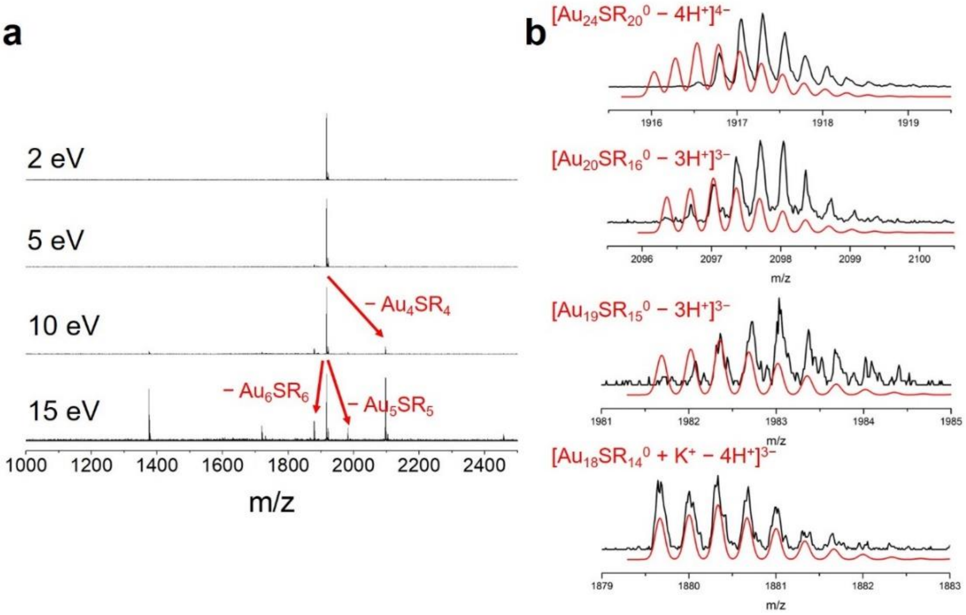

Supplementary Figure 34. MS/MS spectra of $[Au_{24}SR_{20}]^0$. (a) MS/MS spectra of $[Au_{24}SR_{20}]^0$ at different fragmentation energy. (b) Analysis of the corresponding species in MS/MS spectra. The shape change of isotopic patterns is due to the ion filtering in the MS/MS process, while keeping the overall position accurate. Note that the 2nd generation fragmentations and small fragments (e.g., $[Au_4SR_4]^-$) also present in MS/MS spectra and they are not analyzed in detail here.

Oxidative etching of $[Au_{25}SR_{19}]^0$

$$[Au_{25}SR_{19}]^0 + 2RS\cdot + RS^- \rightarrow [Au_{24}SR_{20}]^0 + [AuSR_2]^-$$

$$[Au_{25}SR_{19}]^0 + 2RS\cdot + 2RS^- \rightarrow [Au_{23}SR_{19}]^0 + 2[AuSR_2]^-$$

Formation of long-chain complexes

$$[Au_{18}SR_{14}]^0 + 2RS\cdot + 3RS^- \rightarrow [Au_{15}SR_{13}]^0 + 3[AuSR_2]^-$$

$$[Au_{15}SR_{13}]^0 + 2RS\cdot + 5RS^- \rightarrow [Au_{10}SR_{10}]^0 + 5[AuSR_2]^-$$

Supplementary Figure 35. Oxidative etching reactions in Stage II. Reactions responsible for oxidative etching of $[Au_{25}SR_{19}]^0$ and formation of long-chain complexes were proposed.

Supplementary Table 1. Intermediate species ($N^* > 0$) identified in the growth process of $[Au_{25}SR_{18}]^-$ using CO as reducing reagent.[9]

2 e	4 e	6 e	8 e	10 e
$[Au_{11}SR_9]^0$	$[Au_{15}SR_{12}]^-$	$[Au_{19}SR_{13}]^0$	$[Au_{23}SR_{16}]^-$	$[Au_{29}SR_{20}]^-$
$[Au_{15}SR_{13}]^0$	$[Au_{18}SR_{14}]^0$	$[Au_{20}SR_{14}]^0$	$[Au_{25}SR_{18}]^-$	
		$[Au_{20}SR_{15}]^-$		
		$[Au_{21}SR_{15}]^0$		
		$[Au_{21}SR_{16}]^-$		
		$[Au_{22}SR_{16}]^0$		
		$[Au_{22}SR_{17}]^-$		
		$[Au_{23}SR_{17}]^0$		

Supplementary Table 2. Intermediate species ($N^* > 0$) identified in the growth process of $[Au_{25}SR_{18}]^-$ using $NaBH_4$ as reducing reagent.[10]

2 e	4 e	6 e	8 e	10 e
$[Au_{15}SR_{13}]^0$	$[Au_{16}SR_{13}]^-$	$[Au_{20}SR_{14}]^0$	$[Au_{23}SR_{16}]^-$	$[Au_{29}SR_{20}]^-$
	$[Au_{17}SR_{14}]^-$	$[Au_{21}SR_{15}]^0$	$[Au_{25}SR_{18}]^-$	
	$[Au_{18}SR_{14}]^0$	$[Au_{21}SR_{16}]^-$		
		$[Au_{23}SR_{17}]^0$		
		$[Au_{24}SR_{18}]^0$		

Supplementary Table 3. Species identified in the etching process ($N^* > 0$) of $Au_{25}SR_{18}$ NCs and their presence/absence in the growth process of $Au_{25}SR_{18}$ NCs by using CO or $NaBH_4$ as reducing agent.[a]

<table>
<tr><th></th><th>Species</th><th>Etching</th><th>Growth/CO</th><th>Growth/NaBH4</th></tr>
<tr><td rowspan="8">Stage I</td><td>$[Au_{25}SR_{18}]^{-/0}$</td><td bgcolor="#FFC000"></td><td bgcolor="#FFC000"></td><td bgcolor="#FFC000"></td></tr>
<tr><td>$[Au_{24}SR_{18}]^{0}$</td><td bgcolor="#FFC000"></td><td></td><td bgcolor="#FFC000"></td></tr>
<tr><td>$[Au_{23}SR_{17}]^{0}$</td><td bgcolor="#FFC000"></td><td bgcolor="#FFC000"></td><td bgcolor="#FFC000"></td></tr>
<tr><td>$[Au_{22}SR_{17}]^{-}$</td><td bgcolor="#FFC000"></td><td bgcolor="#FFC000"></td><td></td></tr>
<tr><td>$[Au_{21}SR_{16}]^{-}$</td><td bgcolor="#FFC000"></td><td bgcolor="#FFC000"></td><td bgcolor="#FFC000"></td></tr>
<tr><td>$[Au_{20}SR_{15}]^{-}$</td><td bgcolor="#FFC000"></td><td bgcolor="#FFC000"></td><td></td></tr>
<tr><td>$[Au_{18}SR_{14}]^{0}$</td><td bgcolor="#FFC000"></td><td bgcolor="#FFC000"></td><td bgcolor="#FFC000"></td></tr>
<tr><td>$[Au_{15}SR_{13}]^{0}$</td><td bgcolor="#FFC000"></td><td bgcolor="#FFC000"></td><td bgcolor="#FFC000"></td></tr>
<tr><td rowspan="6">Stage II</td><td>$[Au_{25}SR_{19}]^{0}$</td><td bgcolor="#FFC000"></td><td></td><td></td></tr>
<tr><td>$[Au_{24}SR_{20}]^{0}$</td><td bgcolor="#FFC000"></td><td></td><td></td></tr>
<tr><td>$[Au_{23}SR_{19}]^{0}$</td><td bgcolor="#FFC000"></td><td></td><td></td></tr>
<tr><td>$[Au_{22}SR_{18}]^{0}$</td><td bgcolor="#FFC000"></td><td></td><td></td></tr>
<tr><td>$[Au_{20}SR_{16}]^{0}$</td><td bgcolor="#FFC000"></td><td></td><td></td></tr>
<tr><td>$[Au_{16}SR_{14}]^{0}$</td><td bgcolor="#FFC000"></td><td></td><td></td></tr>
</table>

[a] Presence: yellow; absence: blank.

Supplementary References

1. Ahlrichs, R., Bar, M., Haser, M., Horn, H. & Kolmel, C. Electronic-structure calculations on workstation computers - the program system turbomole. *Chem. Phys. Lett.* **162**, 165 (1989).
2. Grimme, S., Antony, J., Ehrlich, S. & Krieg, H. A consistent and accurate ab initio parametrization of density functional dispersion correction (DFT-d) for the 94 elements h-pu. *J. Chem. Phys.* **132**, 154104 (2010).
3. Tao, J. M., Perdew, J. P., Staroverov, V. N. & Scuseria, G. E. Climbing the density functional ladder: Nonempirical meta-generalized gradient approximation designed for molecules and solids. *Phys. Rev. Lett.* **91**, 146401 (2003).
4. Schafer, A., Huber, C. & Ahlrichs, R. Fully optimized contracted gaussian-basis sets of triple zeta valence quality for atoms li to kr. *J. Chem. Phys.* **100**, 5829 (1994).
5. Andrae, D., Haussermann, U., Dolg, M., Stoll, H. & Preuss, H. Energy-adjusted abinitio pseudopotentials for the 2nd-row and 3rd-row transition-elements - molecular test for Ag2, Au2 and RuH, OsH. *Theoretica Chimica Acta* **78**, 247 (1991).
6. Chen, S. *et al.* The structure and optical properties of the [$Au_{18}(SR)_{14}$] nanocluster. *Angew. Chem. Int. Ed.* **54**, 3145 (2015).
7. Zeng, C. J., Liu, C., Chen, Y. X., Rosi, N. L. & Jin, R. C. Gold-thiolate ring as a protecting motif in the $Au_{20}(SR)_{16}$ nanocluster and implications. *J. Am. Chem. Soc.* **136**, 11922 (2014).
8. Pei, Y., Tang, J., Tang, X. Q., Huang, Y. Q. & Zeng, X. C. New structure model of $Au_{22}(SR)_{18}$: Bitetrahederon golden kernel enclosed by $Au_6(SR)_6$ Au(I) complex. *J. Phys. Chem. Lett.* **6**, 1390 (2015).
9. Luo, Z. T. *et al.* Toward understanding the growth mechanism: Tracing all stable intermediate species from reduction of Au(I)-thiolate complexes to evolution of Au_{25} nanoclusters. *J. Am. Chem. Soc.* **136**, 10577 (2014).
10. Chen, T. K. *et al.* Synthesis of water-soluble $[Au_{25}(SR)_{18}]^-$ using a stoichiometric amount of nabh4. *J. Am. Chem. Soc.* **140**, 11370 (2018).
